# Metabolomic Profiling of Arginine Metabolome Links Altered Methylation to Chronic Kidney Disease Accelerated Atherosclerosis

**DOI:** 10.4172/jpb.S14-001

**Published:** 2015-05-18

**Authors:** Anna V Mathew, Lixia Zeng, Jaeman Byun, Subramaniam Pennathur

**Affiliations:** 1 Division of Nephrology, Department of Internal Medicine, University of Michigan, Ann Arbor, MI, USA; 2 Department of Computational Medicine and Bioinformatics, University of Michigan, Ann Arbor, MI, USA

**Keywords:** Chronic kidney disease, Animal model, Arginine methylation, Atherosclerosis, Asymmetric dimethyl arginine

## Abstract

Atherosclerotic cardiovascular disease is the leading cause of death in patients with chronic kidney disease (CKD), but the mechanisms underlying vascular disease has not been fully understood. As the nitrogen donor in nitric oxide (NO^·^) synthesis, arginine and its metabolic products are integrally linked to vascular health and information. We hypothesized that derangements in this pathway could explain, in part, increased atherosclerotic risk in CKD. We developed a targeted metabolomic platform to profile quantitatively arginine metabolites in plasma by liquid chromatography tandem mass spectrometry (LC/MS). Male low-density lipoprotein receptor defcient (LDLr^−/−^) mice at age 6 weeks were subjected to sham or 5/6 nephrectomy surgery to induce CKD. Subsequently, the animals were maintained on high fat diet for 24 weeks. Targeted metabolomic analysis of arginine metabolites in plasma was performed by isotope dilution LC/MS including asymmetric dimethyl arginine (ADMA), symmetric dimethyl arginine (SDMA), N-mono-methylarginine (NMMA), arginine and citrulline. Although elevated plasma levels of ADMA and SDMA were found in the CKD mice, only higher ADMA level correlated with degree of atherosclerosis. No significant differences were noted in levels of NMMA between the groups. CKD mice had high levels of citrulline and arginine, but ADMA levels had no correlation with either of these metabolites. These fndings strongly implicate altered arginine methylation and accumulation of ADMA, may in part contribute to CKD accelerated atherosclerosis. It raises the possibility that interrupting pathways that generate ADMA or enhance its metabolism may have therapeutic potential in mitigating atherosclerosis.

## Introduction

Coronary artery disease (CAD) and cardiovascular (CV) events associates with chronic kidney disease (CKD) and is the leading cause of death in patients with CKD (>10-fold mortality). Indeed, CV events and mortality are more likely outcome than progression to end-stage renal disease (ESRD) in CKD subjects [1–6]. Cross-sectional studies have demonstrated that the traditional risk factors are only partially predictive of CAD in CKD subjects, implying the presence of additional CKD- specific risk factors [2]. In response to physiologic stimuli, endothelial cells dynamically regulate arterial vascular tone by producing vasodilators and vasoconstrictors. Risk factors for atherosclerosis, such as CKD, interfere with this response, promoting endothelial dysfunction and atherosclerosis. One key regulator is nitric oxide (NO), which is generated from L-arginine by endothelial nitric oxide synthase (eNOS) in the presence of cofactors such as tetrahydrobiopterin. Gaseous NO diffuses to vascular smooth muscle cells and activates guanylate cyclase, which in turn elevates cyclic guanosine monophosphate to promote vasodilation. NO’s antithrombotic nature prevents platelet aggregation, promotes fibrinolysis and decreases smooth muscle proliferation [7].

**Figure 1:**
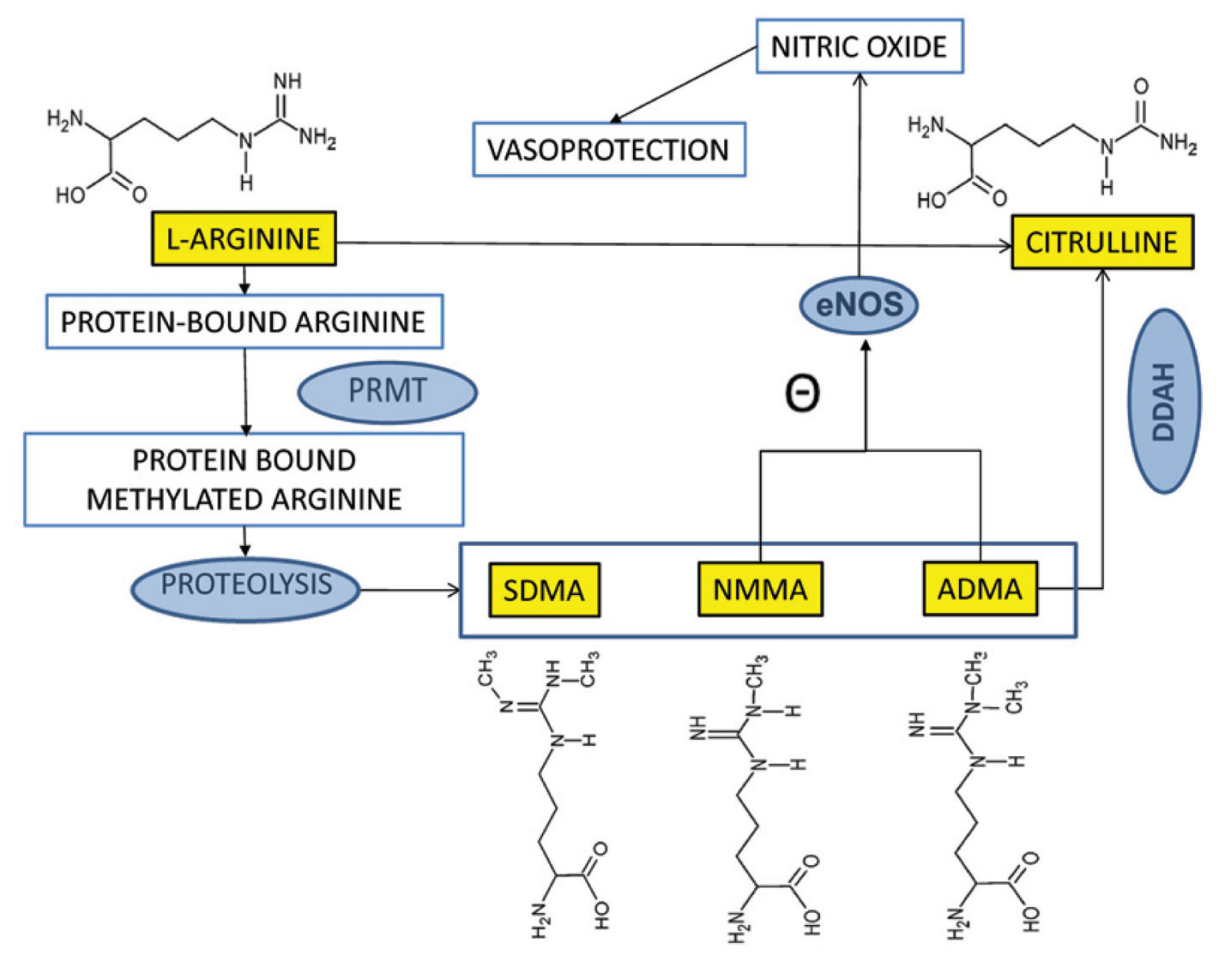
Arginine Metabolic Pathways Arginine residues in intact proteins are methylated by protein arginine methyl transferases (PRMT) in to protein-bound N^G^monomethyl-L-arginine (NMMA), N^G^N^G^dimethylarginine (ADMA) and N^G^N′^G^dimethylarginine (SDMA). Upon proteolysis, free NMMA, SDMA and ADMA are released into circulation. Both ADMA and NMMA are inhibitors of endothelial nitric oxide synthase (eNOS) - which synthesizes Nitric oxide – a potent vasoprotector from free arginine. ADMA is degraded to citrulline by dimethylarginine dimethyl aminohydrolase (DDAH) in the kidneys. The chemical structures of arginine metabolites are depicted.

Asymmetric dimethylarginine (ADMA: N^G^N^G^dimethylarginine) is the naturally occurring dimethylated modification of arginine and is a known inhibitor of NOS. One pathway for producing ADMA is proteolysis of methylated proteins which are formed by protein arginine methyl transferases (PRMT) (Figure 1). Once released into plasma, ADMA can inhibit eNOS and decrease NO bioavailability, causing endothelial dysfunction. The enzyme dimethyl arginine dimethyl amino hydrolase (DDAH) metabolizes ADMA to generate dimethylamine and citrulline [8–11]. DDAH has two isoforms of which DDAH1 is thought be the primary enzyme responsible for ADMA degradation [12–14]. While the proximal tubules of kidneys can reabsorb almost all of the filtered L-arginine, very little ADMA is reabsorbed or excreted into urine. The majority of filtered ADMA is degraded into citrulline and dimethylamine by the renal DDAH as the kidneys have abundant amount of DDAH1. Thus in CKD, loss of DDAH1 activity may limit ADMA breakdown [15]. Symmetric dimethyl arginine (SDMA; N*^G^*N′*^G^*dimethylarginine), a stereo somer of ADMA is also produced by proteolysis following PRMT methylation of protein-bound arginine, but has no NOS inhibitory activity and is renally excreted. N^G^monomethyl-L-arginine (NMMA) is the precursor of both ADMA and SDMA and is a more potent but less abundant NOS inhibitor. The effect of these methylation products on hypertension and cardiovascular morbidity in both CKD and healthy populations is well established [9,16–20]. Human studies have demonstrated that plasma ADMA levels are increased in CKD, hypertension, diabetes, obesity and metabolic syndrome [21–25] and are able to predict CV events [26,27]. Higher ADMA levels also predict cardiovascular events in dialysis patients [28,29], CAD related mortality in pre-dialysis CKD patients [30–32] and associates with CKD progression [18,33]. ADMA is known to promote renal collagen and TGF-β production, thus promoting renal fibrosis and partly explaining the association with CKD progression [34]. While these associative studies raise the possibility that altered arginine metabolism may contribute to atherosclerosis in CKD, a direct link between degree of atherosclerosis and the arginine metabolome has not been previously tested in experimental models of CKD atherosclerosis. In this study, we demonstrate altered arginine methylation is linked to degree of atherosclerosis in a CKD mouse model, raising the possibility of a therapeutic potential of interrupting this pathway in CKD-atherosclerosis.

## Methods

### Reagents and materials

Male C57BL/6 LDLr^−/−^ mice were purchased from Jackson Labs, Bar Harbor, ME. Authentic and isotopically labeled standards were purchased from the following vendors: NMMA, SDMA and D_6_ SDMA (Santa Cruz, CA); D_7_ ADMA (Novachem, Australia); Creatinine, D_3_ Creatinine, Arginine and Citrulline (Sigma-Aldrich); ^13^C_5_-Citrulline and ^13^C_6_-Arginine (Cambridge Isotope Laboratories, MA). All LC reagents were purchased from Sigma Aldrich, St. Louis, MO. Rodent diets were purchased from LabDiet^®^ and high fat diet from Harlan Teklad.

### Mouse models

All animal procedures were approved by the University of Michigan Committee on Use and Care of Animals. Six week old male C57BL/6 LDLr^−/−^ mice were fed LabDiet^®^ standard rodent diet that has 200 ppm cholesterol, 28.5% protein, 13.5% fat and 58.0% carbohydrates by calories. Mice were housed in a climate-controlled, light-regulated facility with a 12:12 hour light-dark cycle and water ad libitum. At age 7 weeks, mice were subjected to either to sham surgery (Control, n=11) or to 5/6 nephrectomy to induce CKD (CKD, n =11). This was accomplished by removing entire right kidney in a first procedure and then subsequent removal of two thirds left kidney by dissection after one week interval. At 9 weeks of age, mice in each group were fed on high fat diet containing 19.5% protein, 40.5% fat, 0.5% cholesterol and 40.0% carbohydrates from Harlan Teklad (TD00243). Murine systolic blood pressure was measured by the IITC Life Science blood pressure system (Woodland Hills, CA) with a highly sensitive photoelectric sensor. The accuracy of measurement was confirmed with five or more successful readings obtained and secured by regular calibration of the pressure transducer. Blood was collected from saphenous veins in living mice with tubes containing dry ethylenediaminetetraacetate (EDTA). Hematocrit was measured by CritSpin^®^ Micro-Hematocrit centrifuge with Digital Hematocrit Reader (StatSpin^®^ Company).

### Analysis of kidney function

Plasma creatinine levels were measured by liquid chromatography electron spray ionization and tandem mass spectrometry (LC/ESI/MS) using an Agilent 6410 MS coupled with 1200 LC system (Agilent, New Castle, DE), as described previously [35]. Briefly, 5 μl of plasma (n=11, each group) and 5 μl of deuterated creatinine (10 pmol/μl) were added into 90 μl of 20 mM ammonium acetate solution, subjected to protein precipitation by 85% acetonitrile. 2 μl of the supernatant was then injected for LC/MS analysis. A hydrophilic interaction chromatography (HILIC) was performed utilizing Luna Phenomenex column (2.1 × 150mm, 3μm; Torrance, CA)) with an isocratic gradient of 85% acetonitrile with 20 mM ammonium acetate for 5 min at flow rate of 0.3 mL/min. The ion transition of the *m/z* 114 to *m/z* 44 for creatinine and *m/z* 117 to *m/z* 47 for D_3_-creatinine was monitored in the multiple reaction monitoring (MRM) mode. The creatinine concentration in each plasma sample was determined by comparing the peak areas of the creatinine and D_3_-creatinine for the above transitions. Blood urea nitrogen (BUN) was measured directly on IDEXX VetTest 8008 chemistry analyzer (Westbrook, Maine) using dry slide technology.

### Analysis of atherosclerosis

At 24 weeks, mice were anesthetized, the thoracic cavity was exposed with a small incision in the right cardiac auricle, and a cannula was inserted into the left ventricle. Through the left ventricle, the animal was perfused with phosphate-buffered saline until the eluent from the right auricle became clear, and then the left ventricle was injected with 3 ml of 10% buffered formalin. Finally, the entire mice were immersed in the fixative at 4°C. Each aortic tree was microdissected to remove adventitial fat and stained with Oil Red O (Sigma) to visualize neutral lipids, pinned on wax plates. The images of the aorta were captured on a digital camera. *En face* plaque quantification was performed with Image Pro software (Media Cybernetics, Bethesda, MD). The lesional areas are represented as ratios between surface area of atherosclerotic lesion stained with Oil Red O to the surface area of the entire aortic tree (n=11, each group).

### Arginine metabolome profiling by LC/MS

The detailed method development and chromatography optimization strategy for arginine metabolomic profiling are discussed in Results. Targeted metabolomic analysis of arginine metabolome in plasma was performed by LC/MS in the positive mode (n=11, each group). Briefly, 20 μL of ethylenediaminetetraacetic acid anticoagulated plasma was spiked with D_7_ ADMA (10pM/sample), D_6_ SDMA (10pM/ sample), ^13^C_6_-arginine (600pM/sample) and ^13^C_5_-citrulline (600pM/ sample). Protein was precipitated with 500μL acetonitrile. 5 μL of the supernatant was subjected to HILIC with a Phenomenex Luna 3 μm, 2 × 150mm column using an Agilent 1200 LC, at a flow rate of 300 μL/min. Solvent A was 10mM ammonium formate and solvent B was acetonitrile with 0.1% formic acid. The column was equilibrated with 95% solvent B and 5% solvent A initially. The gradient was: 95–15% solvent B over 8 min, 15% solvent B for 6 min, 15–95% solvent B for 1 min and then finally 95% solvent B for 10 min.

The eluent from the LC was subjected MS analysis using an Agilent 6410 Triple Quadrupole MS system) connected in series to the LC, equipped with an electrospray source. Positive LC/ESI/MS was performed using following parameters: spray voltage 4000 V, drying gas flow 15 L/min, drying gas temperature 325°C, and nebulizer pressure 40 psi. Flow injection analysis (FIA) using MS^2^ scan was used to optimize the fragmentor voltage and collision energy for arginine, citrulline, NMMA, ADMA, and SDMA. To obtain the best signal-to-noise ratio for quantitation, the most abundant ions from each compound were chosen for use in MRM mode (Table 1). Limits of detection (LOD) were calculated using peak areas corresponding to greater than five times signal to noise ratio. Data analysis was performed with Agilent Mass Hunter Analyst software (Version B6, Agilent, Santa Clara CA). In preliminary studies, we found that the correlation between the peak areas of labelled standards and authentic compounds remained linear and greater than 0.95 irrespective of internal standard concentrations being a log fold higher or lower than the authentic compound concentration for all the arginine metabolites. The amount of isotope labelled standard spiked was subsequently individualized to each metabolite based on roughly 1:1 ratio of what is expected in the physiological range. Ratios of the peak area of metabolites to the spiked isotope labelled compounds were used for quantification since the amount of spike was known for each analyte that was measured. The most abundant fragment ion was chosen as the MRM transitions for each metabolite (Table 1).

### Statistical analysis

Results are given as mean ± SEM. Differences between the groups were considered significant at p<0.05 using the program GraphPad Prism version 6.00 for Windows (La Jolla, California, USA) for the independent t test when comparing characteristics of the different groups. Pearson’s correlation was used to measure the strength of a linear association between two variables using SPSS software for version 22 (SPSS Inc., Chicago, Illinois). A two-sided p<0.05 was considered significant.

## Results

### Development of a targeted arginine metabolome profiling platform by LC/MS

#### Selection of column and LC conditions for optimal separation of the analytes

To select the most appropriate column and chromatography technique, we attempted several different columns with both HILIC and reverse phase to optimize the separation. The columns and LC conditions were based on previous literature. We first examined the Phenomenex phenyl hexyl column (2.0 × 100mm, 3μm, Torrance, CA) with 10mM ammonium formate as solvent A and methanol:H_2_O with 0.1% formic acid (1:3) as solvent B at a flow rate of 0.25 mL/min. The column was equilibrated with 100% solvent A initially and then decreased to 0% from 1–3 min, and then finally 100% solvent B from 3–5 min. This resulted in very broad peak shapes. We then used the Agilent C18 reverse phase column (2.1 × 50 mm, 1.8μm) with H_2_O + 0.1% formic acid as solvent A and acetonitrile with 0.1% formic acid as solvent B at flow rate of 0.3 mL/min. The gradient was 100% solvent A to begin with which was decreased to 0% at 6 min and 100% solvent B was maintained between 4 min and 6 min. The peak areas were sharp but all compounds eluted in the first column volume consistent with non-retention of the analytes to the column. We then tested the Waters Symmetry C18 column (2.1 × 100 mm, 3.5μm, Milford, MA) with 10mM ammonium acetate as solvent A and 100% acetonitrile as solvent B at 0.3 mL/min flow rate. The gradient began with 100% solvent A for 0.5 min, increased linearly between 0.5 min and 5 min to 100% solvent B and stayed at 100% solvent B between 5 and 6 min. This method produced very poor peak shapes. While for the most part peak shapes were acceptable, reverse phase separation resulted non-retention of the analytes as they were eluted within either void or two-column volumes and therefore were sub-optimal. Finally, we attempted Phenomenex HILIC Luna (3 μm, 2 × 150mm column (Torrance, CA)) at a flow rate of 300 μL/min with solvent A 10mM ammonium formate and solvent B acetonitrile with 0.1% formic acid. The column was equilibrated with 95% solvent B and 5% solvent A initially. The gradient was: 95–15% solvent B over 8 min, 15% solvent B for 6 min, 15–95% solvent B for 1 min and then finally 95% solvent B for 10 min. This method produced optimal peaks, retention times and shapes for ADMA, SDMA, NMMA, arginine and citrulline. Therefore, this column and method was chosen for optimizing MS conditions.

#### Optimization of MS parameters

Using FIA of authentic standards, the fragmentor voltage, cell acceleration voltage and collision energy was optimized for each individual compound on an Agilent 6410 triple quadrupole MS. The molecular ion [M + H]^+^ of L-arginine and ^13^C_6_ Arginine are *m/z* 175 and 181 respectively and the loss of *m/z* 105 yields an intense ion at *m/z* 70 *and* 74 respectively. The molecular ion [M + H]^+^ of ADMA, D_7_ ADMA, SDMA and D_6_ SDMA are *m/z* 203, 210, 203 and 209 respectively. The loss of *m/z* 133 is the major fragment ion yielding product ions at *m/z* 70, 77, 70 and 76 respectively. The molecular ion [M + H]^+^ of citrulline and ^13^C_5_ citrulline are *m/z* 176 and 181 respectively and the loss of *m/z* 17 yields fragments at *m/z* 159 and 164 respectively. These transitions form ideal candidates for MRM analysis (Table 1). The limit of detection (LOD) using this methodology was 64 fmol for arginine, 21 fmol for ADMA, 2 fmol for SDMA, 14 fmol for NMMA and 20 fmol for citrulline. The interassay and intra assay variability were 2 to 10% and less than 3% respectively for all compound to internal standard ratios.

**Figure 2:**
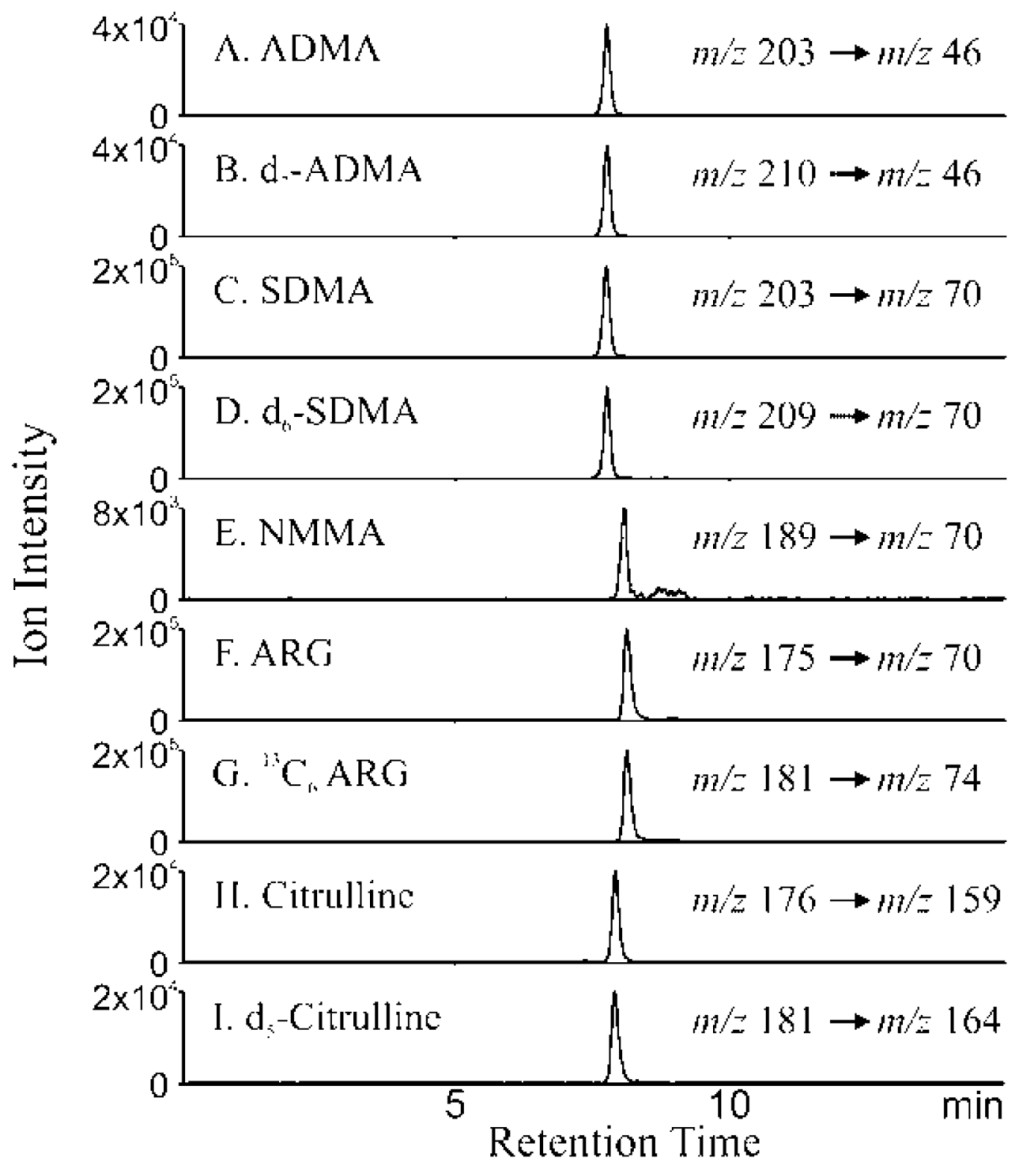
Detection of arginine metabolites by LC/MS Extracted ion chromatograms of ADMA (A), D_7_ ADMA(B), SDMA (C), D_6_ SDMA (D), NMMA (E), arginine (F), ^13^C_6_- arginine (G), citrulline (H) and ^13^C_5_ citrulline (I) are shown. Hydrophilic interaction chromatography (HILIC) of authentic compounds and their internal standards was performed using Phenomenex HILIC column with solvent 10mM ammonium acetate and 100% acetonitrile with 0.1% formic acid. The column was equilibrated with 95% solvent B and 5% solvent A initially. The gradient was: 95–15% solvent B over 8 min, 15% solvent B for 6 min, 15–95% solvent B for 1 min and then finally 95% solvent B for 10 min. The eluent was subjected electrospray ionization mass spectrometry (ESI/MS) and the analytes were detected in the multiple reaction monitoring mode (MRM). X axis is denotes time of elution during chromatographic run and the Y axis represents the ion intensity of the extracted ion chromatogram of the daughter ion following ESI of the analyte in the MRM mode. The MRM transitions that are followed for the individual analyte are depicted with each parent molecular ion to daughter fragment that was monitored.

Figure 2 depicts the extracted ion chromatogram (EIC) for the MRM transitions for ADMA (Panel A), D_7_ ADMA(Panel B), SDMA (Panel C), D_6_SDMA (Panel D), NMMA (Panel E), arginine (Panel F), ^13^C_6_ arginine (Panel G), citrulline (Panel H) and D_5_ citrulline (Panel I) with the optimized HILIC separation. The MRM transitions noted in Figure 1 were utilized for quantitative measurements of arginine metabolome in plasma.

### CKD mouse model has biochemical evidence of CKD and increased atherosclerosis

**Figure 3:**
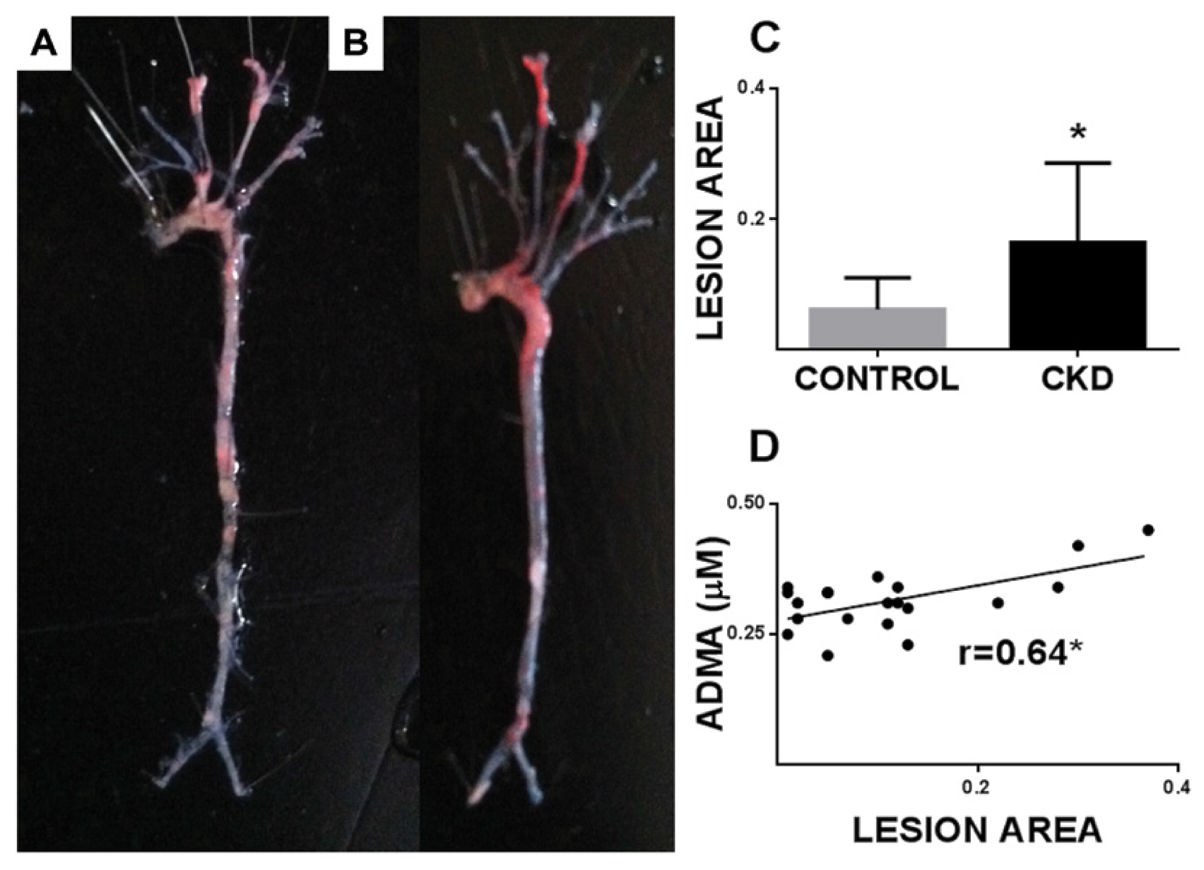
Plasma ADMA correlates with atherosclerotic burden in CKD mice Panel A and Panel B depict a representative *en face* analysis of control and CKD mouse aortic tree following Oil Red O stain respectively. Lesion area was measured as described in “Methods” and represented in Panel C for control and CKD mice (n =11 per group). Plasma ADMA (Panel D) correlated with lesion area using Pearson correlation (*p<0.05).

The CKD mice at 24 weeks had significantly higher plasma creatinine (1.75 ± 0.54 vs. 0.97 ± 0.34 mg/dL; n = 11; p<0.001) and BUN; (44.17 ± 1.79 vs. 28.25 ± 1.17 mg/dL; n = 11; p<0.0001). The CKD mice had significantly lower body weight and hematocrit. The CKD mice did not show significant differences in cholesterol levels, mineral metabolism (calcium, phosphorus and intact parathyroid hormone) or blood pressure (Table 2). We performed *en face* analysis of the entire aorta and stained with Oil Red O to determine lesion area. Figure 3 Panel A and Panel B depict a representative control and CKD mouse aortic arch following Oil Red O stain. The CKD mice had increased atherosclerotic lesion area compared with control mice (0.16 ± 0.04 vs.0.06 ± 0.01; (n=9); p<0.05) (Figure 3: panel C; n=11 per group). The data strongly support induction of CKD as a major factor that accelerates atherosclerosis in this model.

### Altered arginine methylation in CKD mice

**Figure 4:**
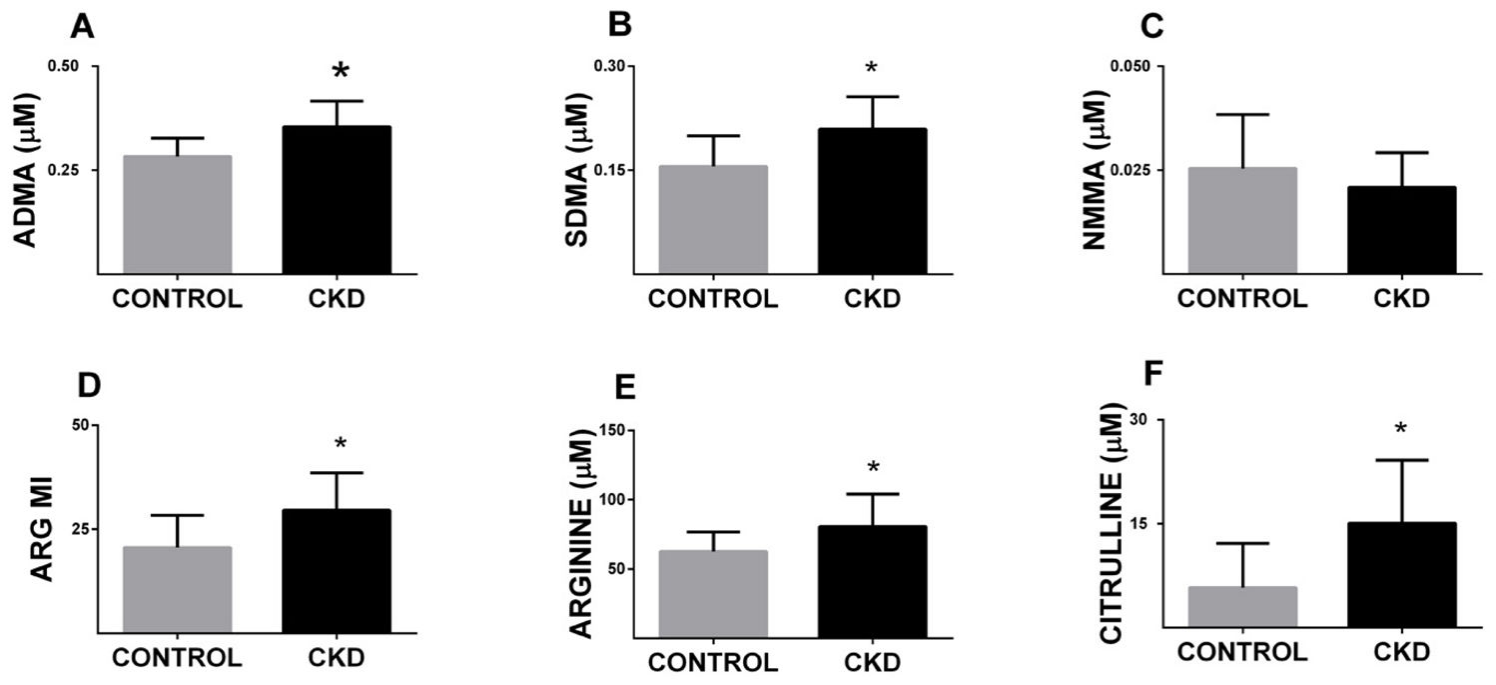
Plasma Arginine metabolome reveals changes in methylation in CKD mice ADMA, SDMA, NMMA levels, arginine, citrulline and arginine methylation index (ARG MI) were measured in plasma in control and CKD LDLr^−/−^ mice by LC/MS. Bars represent mean ± SEM (n= 11 each group). Plasma ADMA (A), SDMA (B), ARGMI (D), arginine (E) and citrulline (F) are significantly higher in CKD mice. NMMA levels are not significantly different (C) (*p<0.05).

Plasma ADMA, SDMA and NMMA levels were measured in plasma collected from the control and CKD mice (n = 11 for each group). The plasma ADMA level was elevated in CKD mice compared to control mice (0.35 ± 0.01 μM vs 0.28 ± 0.01 μM; p<0.01; Figure 4A). Similarly plasma SDMA was elevated in CKD mice compared to control mice (0.20 ± 0.01 μM vs.0.15 ± 0.01 μM; p<0.05; Figure 4B). The values for NMMA were not different between the two groups (Figure 4C). Arginine methylation index, defined as the ratio of dimethylated arginines to the monomethylated precursor (ADMA+SDMA)/NMMA) [36] was significantly higher in the CKD mice, when compared to the control mice (29.55 ± 2.71 vs. 20.64 ± 2.31 p<0.05; Figure 4D). Figure 4 panels E and F depict elevations of plasma arginine (80.66 ± 7.03 μM vs. 62.63 ± 4.25 μM; p<0.05) and Citrulline ((15.07 ± 2.74 μM vs. 5.79 ± 1.92 μM; p<0.05) in CKD mice compared to control mice. These data strongly suggest that CKD alters arginine metabolism, raising the possibility that such alterations may diminish NO bioavailability, contributing to vascular dysfunction.

### Levels of altered arginine methylation products and their substrates correlate with each other

The ADMA levels of both control and CKD mice together correlate with SDMA levels (r = 0.549, p < 0.01) while both arginine and citrulline levels correlate with each other (r = 0.449, p < 0.05; Table 1) using pearson correlation. The substrate for ADMA production- arginine and the by-product of ADMA degradation -citrulline levels do not correlate with ADMA levels (arginine r=0.36 p=0.09; citrulline r=0.24; p=0.58; Table 3). This implies that plasma citrulline is predominantly derived from arginine and not from ADMA. We also performed Spearman correlation analysis between ADMA, SDMA, NMMA and arginine and between citrulline and arginine which showed similar results (Data not shown).

### ADMA levels, but not other arginine metabolites, are strongly associated with atherosclerotic burden

We tested whether arginine metabolites correlated with atherosclerotic burden by comparing levels of the metabolites with lesional area, a measure of degree of atheroma in control and CKD mice by performing Pearson Correlation analysis (Figure 3, panel B). Only ADMA levels correlated with the lesional area (r = 0.64, p<0.01) while SDMA levels did not (r = 0.02, p>0.05). Similarly, NMMA and arginine methylation index did not correlate with degree of atherosclerosis (Data not shown). The association of ADMA to atherosclerosis was stronger in the CKD mice. This data strongly supports the notion that ADMA might directly contribute to degree of atherosclerosis, perhaps by inhibiting eNOS and contributing to decreased NO production.

## Discussion

In this study, we utilized a mouse model of atherosclerosis to address whether alterations in arginine metabolism, in part accounts for increased atherosclerosis observed in CKD. We utilized LC/MS to profile and quantitatively measure arginine and its methylated derivatives in plasma. We established that ADMA, SDMA, NMMA levels and the arginine methylation index are elevated in CKD mice. ADMA levels are directly related to the extent of atherosclerosis in CKD mice, suggesting a central role for altered NO bioavailability in atheroma formation in this model. Finally, we provide evidence that the ADMA elevation is not entirely related to availability of its substrate arginine or correlated to levels of its byproduct citrulline but might be a result of potential enzymatic pathways that lead to formation of methylated proteins or its degradation.

LC/ MS methodology for measurement of these methylated arginines remains the gold standard for accurately measuring these methylated arginines. Arginine and its methylated metabolites are extremely polar and are not well separated with standard reverse phase chromatography. Using HILIC, we are able to achieve optimal separation. Importantly, with the use of tandem spectrometry we are able to measure levels of methylated arginines in the low femtomolar range, which makes it highly sensitive especially for rodent studies where sample availability is limiting. We used four isotopically labeled standards; D_7_ ADMA, D_6_ SDMA, ^13^C_6_ arginine and D_5_ citrulline to accurately determine the instrument response and to account for ion suppression and matrix effects for each specific compound in contrast to previously published methods that use one labeled standard for many compounds [37,38]. This method is the first to use labeled SDMA to accurately determine its concentration independent of ADMA. Using the appropriate isotope labeled standard, this methodology enables a very accurate measurement of the arginine metabolites with minimal interassay and intraassay variability. This method also eliminates derivatization, solid phase extraction and other complicated procedures like ultrafiltration and uses a straight forward protein precipitation process, minimizing sample loss and expense.

The LDL receptor deficient mouse is a well-characterized model for atherosclerosis that develops extensive lesions in aortic root and branches and perivascular system as described previously [39]. The effect of CKD in this mouse model has also been described previously and mainly results in acceleration of the lesions similar to non-CKD mice [40]. The mouse however does not develop coronary atherosclerosis and hence we measured aortic root lesion area as this is the most reproducible measurement. Following 5/6 nephrectomy, the mice demonstrate features of CKD like increased BUN and creatinine. The mice do not have increased blood pressure or changes in the calcium phosphorus metabolism, making CKD the sole variable that could potentially contribute to atherosclerosis. The atherosclerosis in this model is accelerated with high fat diet and similar models in the apoE ^−/−^ background are previously reported [41].

Interestingly, a study by Jacobi et al reported no change in ADMA levels in subtotal nephrectomized mice in apoE^−/−^ background fed on chow diet for 12-months [42]. These findings are in contrast to earlier publications that show increased ADMA levels in CKD models and our current results [30,31]. Also surprisingly in that work, apoE^−/−^ CKD mice did not have higher atherosclerotic burden when compared with DDAH1 overexpressing mice on an apoE^−/−^ background. Over expression of DDAH1 in this mice model however predictably decreased ADMA levels but did not change atherosclerotic lesions. It is likely that these differences are attributable to strain differences, more modest reduction in renal function and diet compared to our study. In our work, CKD mice have accelerated atherosclerosis, manifested as increased luminal lipid accumulation, elevated aortic plaque, necrotic core, fibrosis as well as greater luminal narrowing. Selective alterations of arginine methylation were identified in our study and may in part account for propensity towards increased atherosclerosis in this model. Indeed, the atherosclerotic burden correlated only with ADMA levels, but not SDMA, NMMA or arginine methylation index.

CKD is associated with decreased NO bioavailability either due to decrease in production due to substrate (arginine) limitation, increased levels of ADMA or increased utilization or presence of increased vasoconstrictors [43]. We demonstrate in our work that changes in ADMA in our model is not related to free arginine and citrulline in plasma suggesting that ADMA levels are not entirely just a consequence of increased arginine levels due to decreased renal function. Elevated ADMA levels are probably a result of increased flux in this pathway, partly due to increased methylation of proteins by PRMT, decreased renal excretion and decreased DDAH1 activity. In a previous study, it was demonstrated that ADMA levels are elevated in puromycin-induced CKD rats due to reduced DDAH activity [15]. Studies in mongrel dogs have also revealed microvascular endothelial changes in CKD dogs that was associated with increased ADMA levels and down regulation of DDAH-II enzyme [44]. Activity changes in DDAH might be associated with loss-of-function polymorphisms of a *DDAH* gene, functional inhibition of the enzyme by oxidative stress in CKD and end-stage renal disease, or both [45]. DDAH1 overexpression in the apoE^−/−^ mice in a previous study demonstrated decreased plaque area in a non CKD model associated with lower ADMA levels [46], but this has not been tested in CKD mice. Future studies will need to focus on DDAH isoforms and the effect of DDAH overexpression in relevant CKD models.

In clinical studies higher ADMA levels predicted cardiovascular events when compared to control patients in pre-dialysis subjects [31,47,48]. In a large clinical study, Wang et al demonstrated that arginine methylation index correlated with CVD outcomes [36]. Therefore, we tested the utility of this measurement in our CKD mouse model, but this measure did not correlate with atherosclerotic burden, perhaps due to small numbers in our study. ADMA together with related markers of oxidative stress like myeloperoxidase could potentiate development of atherosclerosis [49]. Renal cyclooxygenase 2 (COX-2) inhibition raises ADMA levels and could explain the increased cardiovascular morbidity of COX-2 inhibitors adding to ADMA’s role in cardiovascular disease [50]. Thus, ADMA together with inflammatory and oxidative markers could play a central role in the accelerated atherosclerotic burden in CKD.

Our model has several strengths. We demonstrate for the first time that in a pathophysiologically relevant model of CKD atherosclerosis altered arginine methylation and a high ADMA levels correlate with atherosclerotic burden. These changes occur even with modest CKD suggesting that these pathways could be relevant in early CKD. The mouse model does not have common associated features of CKD such as hypertension, and abnormal mineral metabolism which make it an ideal model to study the effect of mild CKD alone. The limitations of this work include small numbers in this study and lack of manipulations to alter ADMA levels to show causality with atherosclerotic burden. Manipulation of PRMT, DDAH or the alternate enzyme alanineglyoxalate aminotransferase-2 (AGXT2) [51,52] could provide such direct evidence and future studies need to focus on this issue. Finally, our findings raise the possibility that interrupting arginine methylation pathways could provide a therapeutic avenue for combating CKD-atherosclerosis.

## Figures and Tables

**Table 1:** MS spectral characteristics following collision induced disassociation (CID) of arginine metabolites. Following ESI/MS in positive mode, MS spectrum was obtained for each arginine metabolite. The molecular ion and major daughter ions following CID are represented.

Analyte	Molecular ion (m/z)	Daughter ions (m/z)
ADMA	203	46*, 70, 88, 116, 158
d_6_ ADMA	210	46*, 77, 71, 88, 165
SDMA	203	70*, 116, 88, 165
d_5_ SDMA	209	70*, 77, 116, 94, 60, 49, 30
NMMA	189	70*, 57
Arginine	175	70*, 60, 43
^13^C_6_ Arginine	181	74*, 61, 44, 74
Citrulline	176	159*,70,113
^13^C_5_ Citrulline	181	164*,75,118

* represents the most intense daughter ion that was chosen for MRM transition monitoring for quantification.

**Table 2:** Physiological and Biochemical characteristics of study animals (n=11; each group)

	CONTROL (n=11) Mean ± SD	CKD (n=11) Mean ± SD	p value
Body weight (g)	33.52 ± 1.5	27.39 ± 0.6	<0.01
Blood Pressure (mm Hg)	102.70 ± 4.1	116.30 ± 9.2	NS
Hematocrit (%)	61.0 ± 0.9	52.91 ± 1.2	<0.0001
Serum Creatinine (mg/dL)	0.97 ± 0.34	1.75 ± 0.54	<0.001
Blood urea nitrogen (mg/dL)	28.25 ± 1.17	44.17 ± 1.79	<0.0001

**Table 3:** Pearson Correlation matrix of the arginine metabolite levels of both CKD and control mice (n=11/ group; total =22).

		ADMA	SDMA	NMMA	Citrulline	Arginine
ADMA	Pearson Correlation	1	.549**	.290	.124	.361
	Sig. (2-tailed)		**.008**	.190	.582	.099

SDMA	Pearson Correlation	.549**	1	.017	.158	.073
	Sig. (2-tailed)	**.008**		.942	.482	.748

NMMA	Pearson Correlation	.290	.017	1	.106	.338
	Sig. (2-tailed)	.190	.942		.640	.123

Citrulline	Pearson Correlation	.124	.158	.106	1	.449*
	Sig. (2-tailed)	.582	.482	.640		**.036**

Arginine	Pearson Correlation	.361	.073	.338	.449*	1
	Sig. (2-tailed)	.099	.748	.123	**.036**	

** Correlation is significant at the 0.01 level (2-tailed).

* Correlation is significant at the 0.05 level (2-tailed).

## Abbreviations

ADMA: Asymmetric Dimethyl Arginine (N^G^ N^G^ dimethylarginine)
BUN: Blood Urea Nitrogen
CKD: Chronic Kidney Disease
CAD: Coronary Artery Disease
CV: Cardiovascular
DDAH: Dimethyl Arginine Dimethyl Amino Hydrolase
eNOS: endothelial Nitric Oxide Synthetase
ESI: Electrospray Ionization
EIC: Extracted Ion Chromatogram
FIA: Flow Injection Analysis
HILIC: Hydrophilic Interaction Liquid Chromatography
LC/MS: Liquid Chromatography/ Mass Spectrometry
LDLr^−/−^: Low-density Lipoprotein Receptor Deficient
MRM: Multiple Reaction Monitoring
NMMA: N^G^ Monomethyl-L-Arginine
NO: Nitric Oxide
PRMT: Protein Arginine Methyltransferases
SDMA: Symmetric Dimethyl Arginine (N^G^N′^G^ dimethylarginine)
